# Do beneficiaries’ views matter in healthcare purchasing decisions? Experiences from the Nigerian tax-funded health system and the formal sector social health insurance program of the National Health Insurance Scheme

**DOI:** 10.1186/s12939-017-0711-y

**Published:** 2017-12-28

**Authors:** Ogochukwu Ibe, Ayako Honda, Enyi Etiaba, Nkoli Ezumah, Kara Hanson, Obinna Onwujekwe

**Affiliations:** 1 Department of Health Administration and Management, Faculty of Health Sciences, University of Nigeria, Enugu Campus, Enugu, Nigeria; 2 Health Policy Research Group, College of Medicine, University of Nigeria, Enugu Campus, Enugu, Nigeria; 3 Department of Economics, Sophia University, Tokyo, Japan; 4 Department of Sociology and Anthropology, University of Nigeria, Nsukka Campus, Nsukka, Nigeria; 5 Department of Global Health and Development, London School of Hygiene and Tropical Medicine, London, UK

**Keywords:** Strategic purchasing, Public accountability, Citizen engagement, Community participation, Tax-funded system, Formal sector social health insurance program, National Health Insurance Scheme, Nigeria

## Abstract

**Background:**

Purchasing is a health financing function that involves the transfer of pooled resources to providers on behalf of a covered population. Little attention has been paid to the extent to which the views of that population  are reflected in purchasing decisions. This article explores how purchasers in two financing mechanisms: the Formal Sector Social Health Insurance Programme (FSSHIP) operating under the Nigerian National Health Insurance Scheme (NHIS), and the tax-funded health system perform their roles in light of their responsibilities to the populations.

**Methods:**

A case study approach was adopted in which each financing mechanism is a case. Sixteen (16) in-depth interviews with purchasers and eight (8) focus group discussions with beneficiaries were held. Agency and organizational behavioural theories were used to characterise the purchaser-citizen relationships. A deductive framework approach was used to assess whether actions identified in a model of ‘ideal’ strategic purchasing actions were undertaken in each case.

**Results:**

For both cases, mechanisms exist to reflect people’s health needs in purchasing decisions, including quantitative and qualitative needs assessment, mechanisms to raise awareness of benefit entitlements and allow choice. However, purchasers do not use the mechanisms to effectively engage with and hold themselves accountable to the people. In the tax-funded system, weak information systems and unclear communication channels between the purchaser and citizens constrain assessment of needs; while timeliness of health information and poor engagement practices of Health Maintenance Organisations (HMOs) are the main constraints in FSSHIP. Inadequate information sharing in both mechanisms limits beneficiaries’ awareness of entitlements. Although beneficiaries of FSSHIP can choose providers, lack of information on the quality of services offered by providers constrains rational decision-making and the inability to change HMOs reduces HMO responsiveness to beneficiary needs.

**Conclusions:**

Responsiveness and accountability to beneficiaries are undervalued by purchasers in both financing mechanisms. In the tax-funded system, civil society organisations can facilitate engagement and accountability of purchasers and the people. In FSSHIP, NHIS needs to provide stronger stewardship of HMOs to promote effective engagement with members. Furthermore, the NHIS should introduce mechanisms that allow FSSHIP members to choose their own HMO, which could encourage HMOs to be more responsive to members.

## Background

Purchasing is a key function of health care financing and is the process through which purchasers, on behalf of the population, transfer pooled resources to health care providers in exchange for health care services [1]. That is, purchasers act as agents for people in the purchase of health care services. Accordingly, to fulfil this agency role effectively, it is important for purchasers to ensure there are effective mechanisms in place to determine and reflect people’s needs, preferences and values in purchasing decisions, and to hold themselves accountable to the population for which they are responsible [2].

Many of the studies that examine health care purchasing have emphasised the relationship between purchasers and providers of services by examining contracts and provider payment mechanisms [3, 4]. However, little attention has been given to the roles and responsibilities of purchasers in relation to the people they represent. This article focuses on the purchaser-citizen relationship for two health care financing mechanisms that operate in Enugu State, Nigeria: the tax-funded health system and the Formal Sector Social Health Insurance Programme (FSSHIP) that operates under the National Health Insurance scheme (NHIS). Specifically, the purpose of the article is to examine how the purchaser in each system performs their roles and responsibilities to the population they serve based on a model of ‘ideal’ strategic purchasing actions.

### The tax-funded system

Under the Enugu State’s tax-funded system, the State Ministry of Health (SMoH) purchases and provides public health services for the entire state population. Residents of the state can elect to use these services. The SMoH defines a minimum package of health care services which covers promotive, preventive, and curative care at primary and secondary care levels, and includes services for communicable and non-communicable diseases, child survival, safe motherhood, nutrition, health education, laboratory services and community mobilisation [5]. SMoH also engages a number of private providers to deliver selected services, including mortuary and immunisation services. Services that are not on the SMoH’s package of services are mostly paid out-of-pocket with user fee required at the point of service utilization.

Health services are predominantly funded through general tax revenue, which the State Government allocates to SMoH as part of the State Budget. All government owned primary and secondary providers are involved in service delivery. Although primary health care falls under the purview of the Local Government Area (LGA), SMoH releases resources to Primary Health Centres (PHCs) for vertical programmes and pays salaries of state public servants and some senior level health workers at the LGAs. The LGA, in turn, pays salaries to all other PHC workers.

### The formal sector health insurance Programme (FSSHIP)

FSSHIP is a Social Health Insurance programme established under the Nigerian National Health Insurance Scheme (NHIS). FSSHIP is a mandatory scheme for employees in the formal sector [6]. As of 2012, coverage was estimated to be about 3% of the population of Nigeria. [7]. Beneficiary contributions to the scheme are based on a proportion of earnings and are accompanied by contributions from employers (employers pay 3.25% of the employee’s consolidated salary while employees pay 1.75%). The NHIS pools funds at the federal level through the National Health Insurance Fund (NHIF). NHIS contracts private, for-profit Health Maintenance Organisations (HMOs) to administer the purchasing system and channel resources to providers. Healthcare providers receive capitation payments for primary healthcare services and fee-for-service for secondary services. A mix of NHIS-accredited public and private health care providers are contracted to deliver services under the FSSHIP.

FSSHIP provides a standard benefit package that specifies primary, secondary and tertiary services, with some exclusions for high technology investigations (CT scans, MRI, etc.) and occupational diseases [6]. FSSHIP members are allocated to specific HMOs, but can choose their primary health care providers from an NHIS accredited provider list.

## Methods

This study was undertaken as part of a multi-country study that examined purchasing functions in 10 countries in Africa and Asia [8]. The multi-country study employed a case study approach in which individual purchasing mechanisms were the cases. The Nigerian study selected two mechanisms for examination: the tax-funded system in Enugu state and the Formal Sector Social Health Insurance Programme (FSSHIP). The citizen-purchaser relationship was the unit of analysis for this paper. The study examined whether there was a difference in the citizen-purchaser relationship between the tax-funded system, where both purchasers and providers belong to the same organisation, and the social health insurance system, where there is organisational separation between purchasers and providers. FSSHIP is financed at the federal level but operates at the state level with beneficiaries being federal government employees and other formal sector workers. FSSHIP and the tax-funded system are the main health purchasing mechanisms operating in Nigeria.

### Study setting

The study was conducted in Enugu state in 2014. Enugu, with a population of about 3.9 million in 2013 [9], is one of 36 states in Nigeria and is located in the South-eastern zone with four other states. The South-east is one of six (6) constitutionally recognised geopolitical zones in Nigeria.

States are grouped into zones based on cultural, historical, and political relationships; the states in each zone have broadly similar patterns and levels of development. In Enugu, there are 17 LGAs, 291 political wards, 471 communities and an urban-rural population ratio of 5:12.

### Data collection

Document review, individual in-depth interviews (IDIs) and focus group discussions (FGDs) were used to collect data for the study. For the tax-funded system, six (6) IDIs were held with respondents from the Ministry of Health and six (6) FGDs were conducted with community members from each of the three purposively selected communities (2 FGD per community). One community was selected from each of the three senatorial zones. For the FSSHIP, a total of ten (10) IDIs were held; six (6) with HMOs and four (4) with NHIS employees. Two (2) FGDs (one male and one female group) were held with beneficiaries of the scheme.

The interview respondents were purposively selected to include the key actor groups involved in healthcare purchasing in the two mechanisms. The conceptual framework described in the following section guided the mapping of the key actor groups. FGD participants in the FSSHIP were beneficiaries of the scheme selected from federal institutions operating in the state. In the tax system, FGD participants were male and female community members recruited from the three senatorial zones. At least one participant was a Facility Health Committee (FHC) member. The officer in charge of health facility that operated in the study community assisted in the recruitment of community members.

Initial mapping of actors based on information areas was used to determine the possible number of interviews. However, interviews were discontinued when no new information was obtained from the focus groups [10]. In addition, the tax funded system has wider coverage hence the need to conduct interviews in all three senatorial zones. The FGDs and IDIs were used to assess beneficiary views on key strategic purchasing actions in the relationship between citizens and purchasers.

Both IDIs and FGDs were conducted by members of the research team; a moderator and a note taker attended each interview. Note takers ensured that the key points for each question were covered and that follow-up questions and prompts were explored. FGDs were primarily run in the native language [Igbo] while IDIs were held in English with respondents at liberty to communicate in either of the two languages. Individual interviews and FGDs were audio recorded with the consent of participants. All interviews were transcribed and those in Igbo were translated into English. Transcribers were well versed in both the Igbo and English languages. The moderators and note takers checked the accuracy of the transcripts against the audio recordings.

A review of grey literature including the 2010-2015 Strategic Health Development Plan, and 2014 Health Policy documentation from the Ministry of Health, the Ministry of Finance and the Planning Department. Government records, including the 2013 and 2014 expenditure reports, budget performance reports, health financing policy documents, health sector reports and presentations at meetings, were examined to determine the context for and expected function of purchasing arrangements as described in the policy design.

### Analytical framework

The multi-country study used a conceptual framework that identified principal-agent relationships between: purchasers and providers; purchasers and citizens; and purchasers and the government [11]. Under agency theory, agents have specific roles to play for their relevant principals [12]. The multiple relationship purchasing framework was used to identify a list of ideal strategic purchasing actions which was compared with policy design and actual practice in order to identify key design and implementation gaps [13].

This article focuses on the relationship between citizens and purchasers and considers the actions that purchasing organisations, as agents, are expected to undertake in order to: be responsive to the needs and preferences of the population they cover (principals); accountable to the people; and ensure people access their entitlements [2]. A broad conceptual framework for strategic purchasing was developed to identify key actions that purchasers, in their role as people’s agent, are expected to undertake [8, 14], including:

1. Assessment of the service needs, preferences and values of the population and use of this information to specify service entitlements/benefits;
2. Informing the population of their entitlements and obligations;
3. Ensuring that the population can access their entitlements;
4. Receiving and responding to complaints and feedback from the population; and
5. Allowing people to choose/exit a purchaser and/or provider.

Organisational behavioural theory suggests that, of the above-listed actions, 1, 4, and 5 can be viewed as either ‘voice’ or ‘exit’ mechanisms to influence purchaser decision-making [15]. While ‘voice’ mechanisms, such as public consultation, advocacy groups, complaint mechanisms, and formal representation of people on purchasing boards, − allow people to express opinions; ‘exit’ strategies allow people to choose purchasers and/or providers and express dissatisfaction by leaving a specific purchasing mechanism or changing an assigned provider.

Determination of how a particular purchaser-citizen relationship is functioning involves the examination of voice and exit mechanisms, and mechanisms that hold purchasers accountable to people (action 2 above) and ensure that people’s entitlements are fulfilled (action 3 above) [15].

### Data analysis

Interview transcripts were the primary data analysed. QSR NVivo 10 was used to organize and manage data. A deductive framework approach was used to analyse the data. The study used the list of actions identified above as the ‘theoretical ideal’ and determined whether those actions were reflected in the policy design in each case (i.e. the tax-funded system and FSSHIP) and how the mechanisms functioned in actual practice in order to identify gaps in both the policy design and implementation.

## Results

Using the analytical framework presented in the Methods section, the findings for each purchasing mechanism are presented according to five themes: (1) assessment of the health needs of beneficiaries; (2) ensuring beneficiary awareness of entitlements and obligations; (3) receiving and addressing complaints; (4) ensuring access to entitlements; and (5) strategies for promoting choice and right to exit.

### Assessment of health needs and update of entitlements to reflect needs, preferences, and values of the population

In both mechanisms, two main needs assessment strategies were identified: (1) processes that aim to collect routine epidemiological data on service utilization; and (2) formal engagement with beneficiaries to elicit their needs and preferences for the services they receive.

### Routine health data collection

In the tax-funded system, the MoH collects routine health service data through the Health Management Information System (HMIS). Reports and statistics on services utilized at health facilities are intended to inform health planning and priority setting. However, MoH respondents indicated that routine data are weak and unreliable for determining needs and making meaningful decisions and plans. Data quality and subsequent usage are compromised due to problems associated with the capacity of human resources, the timeliness of collection and completeness of data reporting:

“*The information is not coming when it is due. In many of the [health] centres, some of the information officers are not functioning as required; to get statistical information is a problem, even if you get the information, what about the quality? You can’t be sure of the data you are getting. So, we have been working hard to improve on all those things so that at least it will help us in planning.”* (IDI 6, MoH respondent)

In the FSSHIP, HMOs are expected to compile and transmit patient encounter data to NHIS on a quarterly basis. The data is gathered at the health facility level and is intended to highlight disease burden and utilization rates. According to a respondent from an HMO:

*“There is an encounter data form, which is an instrument we use to collect data on utilization and service delivery. The encounter data is used in various ways. The major thing is for you to ascertain the level of utilization. For instance, do you have up to 13% of enrolees coming to a hospital; is it 3% or 50%? It shows what percentage of the enrolees you have that are coming. It also shows the disease burden in that particular region.”* (IDI 1, HMO respondent)

There was consensus that the required information is not transmitted as designed because many providers do not compile and make these data available. This was partly attributed to the broad engagement of providers with (multiple) HMOs, and larger health facilities, in particular, are unlikely to deliver monthly patient documentation:

*“Some [providers] are complying but many don’t. The problem is that majority of them are from very big hospitals where they have a lot of activities with other HMOs and a lot of things to do [...] But whenever they come, we compile them and send.”* (IDI 4, HMO respondent)

### Engagement mechanisms

Facility Health Committees (FHCs) serve as a means to engage citizens in the tax-funded health system. The FHC is a community-based arrangement that is established to offer a sustainable channel for promoting citizen voice and to encourage greater accountability and responsiveness in the health system [16]. Committee members are selected through community-based democratic processes (voting at an open community meeting); and based on their trustworthiness and stated willingness to work for their community. Committee members are primarily responsible for: regular interaction with health workers at their designated facilities; regular interaction with members of their local community to understand health needs; providing feedback and liaising with the MoH on their community’s health. These committees have been established in most primary and secondary health facilities in the state. Most FHCs carry out their responsibilities in consulting both health workers and community members to identify needs, but health services are often not improved and identified needs remain unmet:

*“They ask questions to know the state of things; after asking those questions, at the end of the day, we won’t see any improvements.”* (FGD 1, beneficiary of tax-funded system)

Problems relating to unclear communication channels between the FHCs and MoH and the bureaucratic protocols involved in meeting policy makers limit FHC performance:

*“What we are talking about is, the protocols that are involved in seeing them [policy makers] are the issue.”* (FGD 6, beneficiary of tax-funded system).

*“What we want is to have an effective avenue for communication. Now that we have laid our complaints, how do we get them to the Ministry of Health or those in authority so that the problems can be solved? That is our problem.”* (FGD 3, beneficiary of tax-funded system).

Although, by design, volunteerism and stewardship to community are emphasized values for FHC committee members, some provisions have been made for minor, non-monetary incentives, such as recognition in the community and, in the past, some non-governmental agencies provided monetary incentives to FHC members [17]. Currently, the absence of formal incentives further constrains active participation by some members of the FHC committee.

*“You know that it’s not easy to pull out a family man to spend hours on free work. If you call him 2-3 times, the next time he’ll tell you that he is on the farm.... So, if the ministry gave him stipend at the end of the month, it would be of great help.”* (FGD 2, beneficiary of tax-funded system)

Under the FSSHIP, HMOs are mandated to conduct quarterly seminars/interactive sessions to educate beneficiaries on the scope of benefit entitlements, rights, and privileges. In addition, these forums provide the opportunity to assess beneficiaries’ satisfaction with services delivered, and receive complaints, suggestions, and recommendations. Most HMOs report conducting these seminars and engaging with beneficiaries to understand their needs, but beneficiaries expressed mixed responses, with the majority suggesting inadequate and limited engagement with HMOs:

*“Let them [HMO’s] frequent wherever they are assigned, and let their [beneficiary] voice be heard. They are the link between the NHIS and the hospitals [providers] and the beneficiary; let them come close, so that we can talk to them. If we have any complaints, let’s have their [telephone] numbers, maybe the official’s [telephone] number so that we can reach them one-on-one. I have not received my NHIS number. But if they have been coming, somehow, I would have got my number. They need to do their work! They need to do their work!”* (FGD 1, FSSHIP beneficiary).

It was also acknowledged that the nature of the engagement does not allow for the views of all to be represented as the forums involve few people from selected institutions:

*“Like I have just said, there are 64 in the office, and I was the only person that came for that meeting. I believe that it would have been okay if others were given opportunity to air their views.”* (FGD 2, FSSHIP beneficiary)

In general, beneficiaries assert that their preferences for service delivery are not met. For example, problems with long waiting times, quality of services, drugs and overall poor treatment of scheme patients when compared to fee-paying patients have been repeatedly expressed but not yet addressed. However, one HMO noted that input from beneficiary engagement was the basis for the revision of the initial benefit package to include some of the services previously on the exclusion list:

*“The comments we get, we try to feedback to NHIS that these are what people are saying. For instance, the issue of Myomectomy - at the commencement, it wasn’t there, that is a fibroid operation. That has been changed. The issue of maternity care, before it was limited to a particular group of people but now it’s open to all women to have four live births irrespective of the number of births they had before they joined the scheme.”* (IDI 6, HMO respondent).

### Mechanisms through which purchasers ensure beneficiary awareness of entitlements and obligations

In the tax-funded system, Enugu State health law recognises the right to health as Government’s obligation to the citizens. The State Patient Rights Charter, an elaboration of the health law, specifies the rights, entitlements, and obligations of patients as service users. Posters indicating the contents of the charter are expected to be displayed in strategic locations at all public health facilities. The charter is intended to stimulate a change in citizen behaviour by provision of adequate knowledge of legal rights. Although posters are displayed in most public health facilities, the information provided has had little impact; patients remain largely unaware of their rights and do not demand that they be upheld:

*“Our awareness of rights is poor. We have what is called Patients’ Rights Charter. We paste it on the wall. But I am almost 2 years here now and I have not gotten a patient who said, “doctor, see this person refuses to explain the cost of this drug to me”; “It is in the charter”; or “This person is supposed to be on duty or is supposed to be attending but he comes to work at 10am.” That’s why I said our rights awareness is still very poor.”* (IDI 4, public provider respondent)

One policy maker observed arrangements were being made to inform the public about the services available at hospitals:

*“In our district hospitals, we now have community mobilization officers working with us. So they will mobilize communities to take-up the health services offered in their community.”* (IDI 4, MoH respondent)

For the FSSHIP, the rights of beneficiaries are stipulated in the scheme’s operational guidelines, including the specification that patients can exercise their rights. Through continuous education, HMOs are required to ensure that new and existing beneficiaries are aware of the procedures for choosing and changing primary healthcare providers, the criteria for assessment of care, and the scope of benefit coverage. These activities are reportedly being carried out by HMOs:

“*We intermittently organize forums. The HMOs are able to use that opportunity to tell their enrolees their rights and privileges, which is their benefit package.”* (IDI 1, NHIS respondent)

However, beneficiary respondents indicated there was limited awareness of the scope of benefits and entitlements among the majority of beneficiaries. The same group suggested that using the media would achieve wider coverage rather than the current, infrequent forums. Limited awareness of rights has often led to providers making unnecessary financial demands from beneficiaries:

*“Many people don’t even know their rights. You get to the hospital and you are expected to pay just 10% for the drugs. Sometimes, if you get to the hospital and you are not aware of that, the people [hospital] will just extort you; take money from you that they are not supposed to. We want a situation where we know…if government has a registry of the services that we are entitled to receive, we want to have copies so that everybody knows how much is required from us [co-payment]. We want to know. And on the issue of drugs, giving us cheap drugs and all that, we also want it addressed.”* (FGD 2, FSSHIP beneficiary).

### Mechanisms to receive and address complaints

Complaint and redress policy in the tax-funded system stipulates complaint lodgement procedures and appropriate actions in the event that grievances are not satisfactorily resolved. The policy further specifies that providers should pay utmost respect to patients’ opinions and rights within the health system:

*“We are trying to inculcate some level of discipline and decorum in the facilities’ staff, so that they handle the patients with care.”* (IDI 9, MoH respondent)

In terms of seeking resolution, many people are not aware of their rights and are reluctant to complain due to a lack of awareness of processes and little confidence to do so:

*“Well, the citizens are not properly informed. In short, they don’t know their rights most of the time. Someone may go to the hospital and he is not properly treated but he finds it difficult to complain. It’s like a cultural issue”* (IDI 4, MoH respondent).

A second redress mechanism that is stipulated in policy is the placing of opinion/complaints boxes at all public health facilities. In practice, many facilities do not have complaint boxes and, where they do exist, they are not considered useful as they are often poorly secured meaning complains do not reach health authorities:

*“If you put your concerns into those boxes, someone may even take it before it gets to the authority. […] It has no key. The one here is like box. It is broken and someone else can have access to anything you put there.”* (FGD 4, beneficiaries of tax funded system)

Similarly, the HMOs are required to install complaint boxes at provider facilities, periodically conduct satisfaction surveys, and establish 24-h help lines to receive and address beneficiary complaints [6]. All HMOs attest to having functional help lines and responses from respondents also affirm that mechanisms do exist for them to express discontent either by phone, face-to-face during meetings or through scheduled visits from officers (staff of agencies/ministries that have been appointed by HMOs to serve as intermediaries between the agency and their HMO). Despite these mechanisms, beneficiaries assert that complaints, especially those relating to provider behaviour and overall service delivery, are seldom addressed and feedback rarely provided by HMOs:

“*They [HMOs] encourage us to complain about bad treatment and things like that, but after that what happens? Nothing, because we have made series of complaints, but the thing stops there because when you go back to your provider, they still mete out the same treatment.”* (FGD 2, FSSHIP beneficiary)

The main reason reported for poor response to complaints is the limited competition for beneficiaries among HMOs and over-centralization of decision-making by NHIS - most decisions are taken at the national level and take time to be enacted at the community level.

### Mechanisms for purchasers to ensure the population can access their entitlements

In the tax-funded system, primary and secondary public providers are set up and managed by the government and members of the public can directly access care from these providers according to need and ability to pay stipulated fees. Assessment of secondary services is based on referral. The MoH tries to regulate user fees and drug charges so that accessing health services remains affordable to patients. The provision of free MCH services for pregnant women, and children under-5 is a further measure to ensure access for vulnerable groups. A district-based health system (DHS) was implemented to decentralize health services and resources to ensure access to services by directly linking primary and secondary care in each locality. The DHS was expected to meet the infrastructural and human resource redistribution needs by establishing, at a minimum, a primary health facility (PHC) in every ward, while also strengthening referral lines [5]:

“*There is no part of this state that is excluded from this district health system… Some communities have health facilities within them but the ones that don’t were linked to a facility that is closest to them.” (IDI 2, MoH respondent)*

The implementation of the DHS has improved construction of health facilities, especially primary care facilities, in many previously under-served areas. However, there have not been corresponding increases in human resources, health services and the availability of drugs and medical equipment:

*“We are handicapped in so many areas, not just the personnel, but also in the supply of drugs and the other equipment, so the services are not up to the standards that are expected.”* (IDI 1, MoH respondent)

To access health services, HMOs register beneficiaries and issue identification cards. Some beneficiaries reported protracted delays in obtaining registration cards from NHIS. Accessing referral services requires pre-treatment authorization from the relevant HMO within 24 h of the request from the provider facility. Beneficiaries expressed concerns about recurring delays in authorization of referrals by HMOs, which often prevented patients from receiving referral services:

*“There was a day I was there [at hospital] till evening and I didn’t get the go ahead from the HMO, and they [provider] asked me to go [home]. Assuming I had an ailment that could kill me, then I’ll not get help...I don’t understand the reason.”* (FGD 3, FSSHIP beneficiary)

Other barriers to accessing necessary care include the limited availability of NHIS approved drugs, leading to frequent out-of-pocket payment for drugs; and poor treatment of scheme patients compared to fee-paying patients.

“…*NHIS patients are like the riffraff, poor people, the common masses, the nobodies. And so any time you come in there under the platform of NHIS, they look down on you, you don’t get the attention you require.”* (FGD 2, FSSHIP beneficiary).

### Existence of choice and exit strategies

While beneficiaries of the tax-funded system can decide whether to seek publicly-provided care, the mandatory nature of the FSSHIP limits the scope of beneficiaries to exit the scheme if they are federal civil servants. Furthermore, staffs from federal ministries, departments, and agencies (MDAs) are assigned to certain HMOs and beneficiaries are not allowed to switch HMOs, even if they are dissatisfied with that HMO. Respondents were of the view that absence of choice of HMOs has led to a nonchalant attitude towards performing mandated tasks for beneficiaries by HMOs:

*“If we had the chance to change HMOs, they would try to find out what we need. So am suggesting that [allowing beneficiaries to change HMOs] so that the establishment can move en masse.”* (FGD 1, FSSHIP beneficiary)

Beneficiaries are free to choose their own primary provider and change providers after an initial six (6) month period. However, beneficiaries indicated that no criteria have been defined by NHIS to help them to gauge the performance of healthcare providers or of service quality. Beneficiaries’ opinions of providers are largely based on discussions with other beneficiaries, and such judgements are sometimes made using incorrect or unsubstantiated information:

“*I just changed from Hospital A to Hospital B; but I just noticed that hospital A was even better than the place I’m at now. So, I’m just on the high sea, I do not know what to do again.”* (FGD 2, FSSHIP beneficiary).

## Discussion

This study examined the mechanisms used to reflect the needs and preferences of beneficiaries in two healthcare financing mechanisms: the Nigerian tax-funded system and the FSSHIP. It also compared how these financing arrangements function in practice in light of an “ideal” strategic purchasing model. The study found that the policy includes various mechanisms to allow purchasers to undertake strategic purchasing actions. These mechanisms include the use of specific strategies to assess the health needs of beneficiaries, raise awareness of beneficiaries’ rights and entitlements, address beneficiary concerns, and ensure access to health services. However, the strategic purchasing mechanisms operating under the tax-funded system are not effectively implemented and/or managed, and variations in how the strategic purchasing mechanisms are implemented were observed in the FSSHIP. The root causes of implementation gaps vary for the two healthcare financing systems.

In both mechanisms, deliberate design of institutional arrangements is required for mechanisms that allow people to make their opinions known (voice mechanisms) to be effective. The design should include clear channels of communication between health system users and decision-makers, with user-responsive management arrangements also implemented [18]. In the tax-funded system, voice mechanisms relating to needs assessment, member/community engagement devices and complaint systems that enable people’s needs and preferences to be reflected in purchasing decisions all operate independently and are not specifically integrated into the design and review of benefit entitlements. Consequently, benefits do not necessarily reflect the expressed needs and preferences of those who they are designed to help. Effectively engaging and reflecting the needs of beneficiaries in purchasing decisions remains a weakness of the FSSHIP. Voice mechanisms for engaging with beneficiaries and addressing complaints are supposed to be implemented by HMOs and the NHIS is supposed to oversee the implementation of these mechanisms. However, individual HMOs have not implemented these mechanisms according to the FSSHIP guidelines. Consequently, beneficiary engagement mechanisms are rarely robust or sufficiently inclusive to achieve intended outcomes.

A major cause of the implementation gaps in voice mechanisms under the tax system is weak information systems for the generation and transmission of quality, accurate and timely information for use in planning and decision-making. Ineffective community engagement processes and unclear lines of communication in the processes have widened the communication gap between purchasers and citizens and reduced the ability of voice mechanisms to achieve their intended purpose. Under the FSSHIP, implementation gaps are mainly associated with poor beneficiary engagement processes. The poor processes are due to a lack of coordination and inadequate supervision of the HMO practices by NHIS, which has resulted in HMOs neglecting their roles and responsibilities to FSSHIP members.

Raising community and beneficiary awareness of entitlements appears to be undervalued in the tax system. Voice mechanisms function on the existence of informed users with some level of power to influence [15], however the tax system does not include this group. The patient charter has received limited attention from MoH and has not been implemented in such a way that it achieves its intended purpose; beneficiaries have limited awareness of rights and lack of power to influence purchasing decisions. Under the FSSHIP, systems for increasing member awareness of benefit entitlements are specified in the policy design. The contract between HMOs and NHIS specifies that HMOs must implement such systems however, implementation of these tools has not occurred as intended because poor engagement mechanisms also weaken information exchange resulting in low levels of awareness of entitlements by program beneficiaries [19].

Community accountability mechanisms are intended as useful tools for enhancing purchaser accountability and responsiveness while also empowering citizens. The tax-funded system uses the FHCs as an accountability mechanism to communities. In practice, the blurred lines of communication and feedback between the FHCs and the MoH weaken mutual accountability. The literature indicates that properly functioning committees exist in Nigeria and elsewhere to manage facility resources, identify population needs, improve community participation, address complaints and manage overall quality management [16, 20, 21]. The results from this study corroborate the literature but also indicate the main constraints to FHC performance are issues associated with incentives, clarity of roles, and proper communication channels. While a contractual agreement exists between the HMOs, healthcare providers and NHIS in the FSSHIP, the roles of citizens appear to be downplayed due to the absence of explicit contracts and mechanisms through which beneficiaries can directly hold HMOs accountable.

Under the tax-funded system, the implementation of DHS contributed to the proliferation of healthcare facilities in all geographic localities, but shortages and geographic imbalances in the distribution of human resources in the public sector constitute a significant barrier to access. Consequently, many people are still unable to access necessary healthcare services, especially in under-served areas. Under the FSSHIP, the self-serving behaviour of some HMOs can and do constrain access to care, particularly for secondary services which require authorization by HMOs prior to use. The often deliberate efforts by some HMOs to control the volume of service utilization can have significant negative impacts on beneficiaries when they are denied their entitled healthcare or made to contribute out-of-pocket payments [22].

Purchasing mechanisms can include strategies that allow beneficiaries the right of exit and choice. When faced with a loss of customers, increased competition among providers and purchasers can bring improvements in service quality [18]. While there are no explicit exit mechanisms for beneficiaries in the tax-funded system, the health system generally allows exit when users decide to use non-public sector facilities and services. Although the FSSHIP allows choice of primary care providers, a lack of information on the quality of healthcare from different providers limits the ability of members to make rational decisions on the choice of healthcare providers. Conversely, the absence of choice of HMOs for FSSHIP members has created little incentive for competition among HMOs and has weakened the ability of FSSHIP members to compel purchasers to be responsive to their health needs.

For purchasing to support progress towards the achievement of health system and universal coverage goals, purchasers must recognise the need to actively engage the citizens in determining the right combination of services and align resource allocation to health needs. Improved mutual accountability between purchasers and populations they cover  is necessary for increased citizen voice and purchaser responsiveness [11]. While the introduction of strategic purchasing measures remains a challenge, some experiences can be drawn from other country practices. Thailand’s Universal Coverage Scheme (UCS) provides some lessons on how strategic purchasing can evolve over time given the right prerequisites and enabling environment [23].

A limitation of this study is that the two financing mechanisms examined target different groups of the population which may affect the comparison of the relationship between purchasers and beneficiaries between mechanisms. The findings should be interpreted in this context. The two financing mechanisms were selected because they represent the two largest healthcare financing arrangements operating in the state, providing the opportunity for governments to learn important lessons for health system reform and universal health coverage.

## Conclusions

Both the tax-funded system and the FSSHIP include mechanisms that allow purchasers to reflect people’s needs, choices, and preferences in purchasing decisions. The mechanisms include quantitative and qualitative assessment of health needs, mechanisms to raise awareness on benefit entitlements and mechanisms that allow choice. However, purchasers are not using the mechanisms effectively to engage and hold themselves accountable to people they represent. The MoH, HMOs and NHIS appear to give little value to their responsibilities to the people, which is the root cause of the implementation gaps in the voice mechanisms described in policy. Under the FSSHIP, the NHIS’s lack of stewardship over the HMOs, who undertake purchasing administration, has resulted in variations in how HMOs have implemented voice and accountability mechanisms. In addition, under FSSHIP, the inability of members to choose HMOs does not encourage HMOs to be responsive to the needs and preferences of members.

Public purchasers should re-evaluate and strengthen their awareness of their roles and responsibilities to the people they represent, and be more responsive and accountable to the people. Civil society organisations (CSOs) have a role to play in empowering people to make their opinions known and facilitate purchaser engagement with the people. Furthermore, under the tax-funded system, as the current definition of services provided in the public sector is based on monitoring information from health facilities, information systems must be strengthened and the accuracy and timeliness of information improved.

The NHIS should be mindful of the consequences of being unable to meet FSSHIP members’ needs and preferences – the scheme currently suffers from low uptake rates and does not receive sufficient resources from member contributions to cover operating costs. Alternative strategies for improving beneficiaries’ understanding of entitlements are necessary to improve participation. The NHIS should also provide stronger stewardship to HMOs and encourage HMOs to engage with and become accountable to their members. Furthermore, the NHIS should introduce mechanisms that allow FSSHIP members to choose HMOs, facilitating HMO responsiveness to members.

## Abbreviations

DRF: Drug Revolving Fund
FHC: Facility Health Committees
FSSHIP: Formal Sector Social Health Insurance Programme
HMO: Health Maintenance Organisations
IDI: in-depth interviews, FGD, focus group discussion
NHIF: National Health Insurance Fund
NHIS: National Health Insurance scheme
SMoH: State Ministry of Health
